# Tumor Cell Plasticity and Angiogenesis in Human Melanomas

**DOI:** 10.1371/journal.pone.0033571

**Published:** 2012-03-19

**Authors:** Daniela Mihic-Probst, Kristian Ikenberg, Marianne Tinguely, Peter Schraml, Silvia Behnke, Burkhardt Seifert, Gianluca Civenni, Lukas Sommer, Holger Moch, Reinhard Dummer

**Affiliations:** 1 Institute of Surgical Pathology, University Hospital Zurich, Zurich, Switzerland; 2 Biostatistics Unit, Institute of Social and Preventive Medicine, University of Zurich, Zurich, Switzerland; 3 Institute of Anatomy, University of Zurich, Zurich, Switzerland; 4 Clinic of Dermatology, University Hospital Zurich, Zurich, Switzerland; The University of Queensland, Australia

## Abstract

Recent molecular studies provide evidence for a significant transcriptional plasticity of tumor cell subpopulations that facilitate an active contribution to tumor vasculature. This feature is accompanied by morphological changes both *in vitro* and *in vivo*. Herein, we investigated the morphological plasticity of tumor cells with special focus on vasculogenic mimicry and neovascularisation in human melanoma and mouse xenografts of human melanoma cell lines. In melanoma xenograft experiments, different vessel markers and green fluorescent protein expression were used to show how melanoma cells contribute to neovascularization. Additionally, we analyzed neovascularization in 49 primary melanomas and 175 melanoma metastases using immunostaining for blood (CD34) and lymphatic (D2–40) vessel-specific markers. We found significantly more lymphatic vessels in primary melanomas than in melanoma metastases (p<0.0001). In contrast to the near absence of lymphatic vessels within metastases, we found extensive blood micro-neovascularization. Blood micro-neovascularization was absent in micro metastases (less than 2 mm). A significant inverse correlation between Glut-1 expression (implying local hypoxia) and the presence of microvessels indicates their functional activity as blood vessels (p<0.0001). We suggest that the hypoxic microenvironment in metastases contributes to a phenotype switch allowing melanoma cells to physically contribute to blood vessel formation.

## Introduction

Melanoma is a highly aggressive neoplasm. Even very thin primary tumors (Breslow tumor thickness <1 mm) may seed metastases and precipitate rapid death [1], [2]. Several cancer studies, including melanoma, have identified primary tumor lymphangiogenesis and its predictive relevance for lymph node metastasis [3], [4], [5], . Interestingly, animal tumor models for melanoma and breast cancer have revealed vasculature reorganisation in regional lymph nodes prior to invasion by metastatic cancer cells [7], [8], [9], [10].

The introduction of high through put assays such as gene expression arrays have offered new tools for cancer classifications including melanoma. [11]. Recently, we have demonstrated two opposing gene expression profiles in melanoma short term cell cultures associated with proliferative or invasive cell phenotypes and provided evidence for phenotype switching favouring disease progression [12], [13]. This transcriptional plasticity is accompanied by morphological changes *in vitro*, and involves many factors governing angiogenesis. In 1999, Maniotis and co-workers introduced the concept of vasculogenic mimicry (VM) in melanoma [14]. He defined VM as melanoma cells with endothelial-like morphology embedded in a PAS and collagen IV positive extracellular matrix. The matrix with endothelial-like melanoma cells forms loops or networks which may be solid or hollow. VMs have been seen in additional malignant tumor types including breast, liver, prostate and other cancers [15], [16], [17], [18]. The presence of VMs have been associated with a poor clinical outcome [14], [19], [20], [21]. Furthermore, mosaic blood vessels in which both endothelial cells and tumor cells form the luminal surface have been described for both melanoma and colon cancer [22], [23], [24]. Most recently, sophisticated investigations have shown that tumor cells directly participate in blood vessel formation [25], [26].

In the present study we investigated melanoma metastases for neovascularization in both human biopsies and mouse xenografts of human melanoma cell lines. We found VMs and a tremendous blood micro-neovascularization in human biopsies as well as in xenografts. In the mouse xenografts of human melanoma cell lines we showed melanoma cell participation in capillary formation.

## Materials and Methods

### Tissue microarray analysis

Immunohistochemistry was performed on three different tissue microarrays (TMAs) representing a total of 49 primary melanomas and 127 melanoma metastasis. The metastasis array included 21 lymph node and 106 organ metastases. Construction of TMAs was performed as previously described [27]. Furthermore, in order to avoid sampling error immunohistochemistry was performed on whole tissue sections of 35 lymph node and 13 organ metastases. Approval for the use of melanoma TMAs and melanoma metastases was obtained from the official ethical authorities of the Canton Zurich. The consent statement was written (StV 16-2007, permission submitted in the supplement).

### Immunohistochemistry

Immunohistochemical stainings on TMAs and melanoma metastases were performed using an automated immunostainer (Ventana Medical Systems, Tucson, AZ, USA), utilizing the antibodies anti-D2–40 (Dako A/S, clone D2–40, dilution 1∶50, pretreatment: CC1-buffer for 90 min./98°C), anti-CD34 (Novocastra, NCL-END, dilution 1∶60, w/o pre-treatment), detection was performed with Ultra View-HRP-Kit on Ventana Benchmark.

PanMelanoma Cocktail (Biocare Medical, clone HMB-45+M2-7C10+M2-9E3, dilution 1∶50, pre-treatment: CC1-buffer for 20 min/98°C), detection was performed with Ultra View-AP Kit (substrate: New-fuchsin) on Ventana Benchmark. Anti-Glut-1 (Chemicon, AB1341, dilution 1∶1000, pre-treatment: CC1-buffer for 20 min/98°C) and anti-MECA32 (PharMingen, #553849, dilution 1∶100, pre-treatment: Leica buffer H2 for 40 min/98°C) detection were performed with Refine-HRP-Kit on BondMax from Leica. Collagen IV (BMA Biomedicals AG, dilution 1∶30, pre-treatment: Ventana protease 1/12 min.) Detection was performed with iVIEW-HRP-Kit on Ventana Discovery, using secondary antibody Donkey anti-Rabbit biotinylated, Jackson 711-065-152, diluted 1∶80). Substrate for all HRP-Kits was Diaminobenzidin (DAB). Counterstain was done with Hematoxylin from the Immunostaining-Kits

### Human tumor cell isolation and xenotransplantation

Studies described in this publication were performed according to Swiss Animal Welfare laws and specifically as described in animal licenses No. 77/2008 (Int. 3816) issued and approved by the Cantonal Veterinary Office (official state office), of the Canton of Zurich (permission submitted in the supplement). The approval was signed by Mrs. Dr. Simone Gilg, scientific board deputy and responsible for the review board of the Cantonal Veterinary Office, of the Canton of Zurich. The approval ID/permit number is 2008077.

The melanoma cell line (M010308) and the melanoma cell lines used for chromosome 17/HER-2 probe were obtained from consenting patients, according to the guidelines of the University Hospital of Zurich. The permission to produce cell cultures from fresh tumor tissue was given from the cantonal ethic commission in Zürich. The consent statement was written (EK-No. 647, see attached permission in the supplement). Melanoma cell lines used for the chromosome 17/HER-2 probe are additionally described in more detail in a recent publication [28]. HEK293 cells were bought from GenHunter (www.genhunter.com; cat. number Q401).

In order to demonstrate mosaic microvessels 2 xenotransplantations with male/female constellation were established as follows: a male melanoma cell culture (M010308) established 4 years ago which went through at least 20 passages, which was frozen and re thawed several times and finally was used for xenotransplantations in female athymic nude mice. The cell line (M010308) was proven to be negative for CD31 and CD34 by immunohistochemistry (data not shown). From the melanoma culture, a total of 3×10E6 cells were injected into both flanks of 8-week-old female athymic nude mice. Mice were kept in individually ventilated cages for a maximum of 75 days postinjection. Tumour volume was measured using vernier calipers once every 1 or 2 days during the linear growth phase. If at least one xenograft tumor reached 1 cm^3^, the mouse was sacrificed and the tumors were investigated with the y-chromosome probe.

In addition using melanoma patients cell line [28] 4 xenotransplantations were established in the following way: Immediately after surgical resection, solid metastatic lesions were dissociated into single-cell suspensions using HBSS (without Ca^2+^ and Mg^2+^, Invitrogen) containing Collagenase III (1 mg/ml, Worthington Bioch.) and Dispase (0.5 mg/ml, Roche). Incubation at 37°C for 1 h with concurrent mincing allowed complete digestion. The resulting cell suspension was filtered through 40 µm nylon mesh and single cells were harvested. Afterwards the cells were cultured in neuro-sphere conditions and passed 8 times. Due to the limited amount of cells, patient tumor xenografts were established by subcutaneous injection of dissociated bulk melanoma cells into nude BALBc/Swiss mice. Resulting patient tumor xenografts were harvested, dissociated as described above. Thereafter tumor cells were labelled with the neuroectodermal marker p75, sorted by FACS and again injected into nude athymic mice. The resulting tumors were investigated by chromogenic in situ hybridization for the human HER-2 probe localized on chromosome 17.

### *Fluorescence In situ* hybridization (FISH) combined with Fluorescence Immunphenotyping and Interphase Cytogenetics (FICTION)

FICTION simultaneously enables to perform cytogenetic investigation by FISH and immunophenotyping by fluorescence marked immunohistochemistry (FICITION = **f**luorescence **i**mmunophenotyping, and **i**nterphase cytogenetics as a **t**ool for the **i**nvestigation **o**f **n**eoplasms)

### Human chromosome 17/HER2 probe and MECA32

Immunohistochemistry on BondMax (Leica), detection of primary Rat anti-Mouse panendothelial MECA32 (Pharmingen Becton/Dickinson, dilution 1∶100) with Refine HRP-Kit (Leica) and Link-antibody Rabbit anti-Rat (Jackson, dilution: 1∶150). Antigen-Retrieval: HIER buffer2 (Leica) for 40 min, Chromogen DAB, without counterstain. Followed by FISH with HER-2- and centromeric probe for chromosome 17 (ABBOTT Molecular), according to the manufacturers guidelines. Counterstain with DAPI.

### Human X/Y-Chromosome/CD31 (FICTION)

FISH analysis for CEP X (DXZ1) Spectrum Green/CEP Y (DYZ3) Spectrum Orange Probe (ABBOTT Molecular) was performed according to the manufacturers' protocol. FISH procedure was followed by the detection of human endothelial cells: Mouse anti-human CD31 (DAKO, dilution 1∶10) was coupled with a fluorescence labeled secondary antibody: Goat anti-mouse Alexa488 (Molecular Probes, dilution 1∶100), counterstain with DAPI (Incubation at room-temperature for 30 min each).

A cell was defined as human-derived as follows: on confocal microscopy, the nucleus had to present typical endothelial morphology, being positive for the Y-probe, and the CD31 positive cytoplasm had to be fully discernible.

### Human ALU-repeat probe and CD31

All procedures were done on BondMax (Leica) with Kits and solutions from Leica according to the manufacturer's guidelines.

Epitope-retrieval was performed with Bond heat-pre-treatment-buffer 2 for 20 min. at 100°C. Immunohistochemistry was done with rabbit anti-CD31 (Abcam Ltd. Ab28364 dil. 1∶100) and Bond™ Polymer Refine Red Detection (Leica) without nuclear staining. Endogenous biotin was subsequently blocked with Avidin-Biotin-Blocking Kit (CellMarque, Cat. 928B-02) and in-situ-hybridization was performed using enzyme pretreatment (Leica, Enzyme) followed by the DNA-hybridization with a biotinylated human ALU-repeat DNA_positive-control-probe (Leica, Cat. PB0682) according to the manufacturer's protocol. Bound probes were detected with a rabbit anti-biotin antibody (Bethyl Cat. A150-109A, dilution 1∶1500) and with donkey anti-rabbit Dylight488 (Jackson, Cat. 711-486-152, dilution 1∶1000). Slides were mounted with Vectashield Hard-Set Mounting Medium with DAPI (Vector Laboratories, H-1500). The CD31-specific fast red color precipitate was detected in the red and the human DNA-specific ALU-repeat in situ hybridization in the green fluorescence channel, respectively.

### GFP (Green fluorescent protein)

Immunohistochemistry on BondMax (Leica) with Refine-DAB-Kit including counterstain. Epitop-retrieval with Bond Heat-pre-treatment-buffer 2 for 30 min. Primary antibody Chicken anti-GFP (Abcam ab13970, 1∶2000), secondary antibody Rabbit anti-Chicken Y (Abcam ab6753, 1∶300).

### Fluorescence Imaging

Confocal image acquisition was done using sequential mode on a Leica TCS SP5 confocal laser scanning microscope (Mannheim, Germany). Co-localization studies were conducted using IMARIS software (Bitplane, Zurich, Switzerland).

### Melanoma cell infection with GFP-labelled lentivirus

Freshly isolated melanoma cells were injected into nude athymic mice. Patient tumour xenografts were harvested and cells dissociated as described above. Some of those cells were cultured in neuro-sphere condition and passed 8 times. At the 8th passage the cells were infected with a GFP driving virus.

GFP-expressing lentivirus was produced by transient four-plasmids cotransfection into HEK 293 [29] with VSV-pseudotyped third-generation lentiviral vectors (LV) by using calcium phosphate method. Supernatants were collected 48 hours after transfection, filtered through a 0.22 mm membrane [30] and transferred to target cells. Resulting GFP-tagged tumor cells were checked by FACS and subcutaneously injected as described above.

### Statistical analyses

CD34 and D2–40 expressions are presented as median with range and compared between different patient groups using the Mann-Whitney and the Krustal-Wallis test. Glut-1 expression was compared using Fisher's exact test. Differences between CD34 and D2–40 expressions were compared using the Wilcoxon signed rank test. P-values below 0.05 were considered as significant. SPSS 16.0 for Windows (SPSS Inc., Chicago, IL) was used for statistical analyses.

## Results

### Primary melanomas show a higher lymphatic vessel density than melanoma metastases

**Figure 1:**
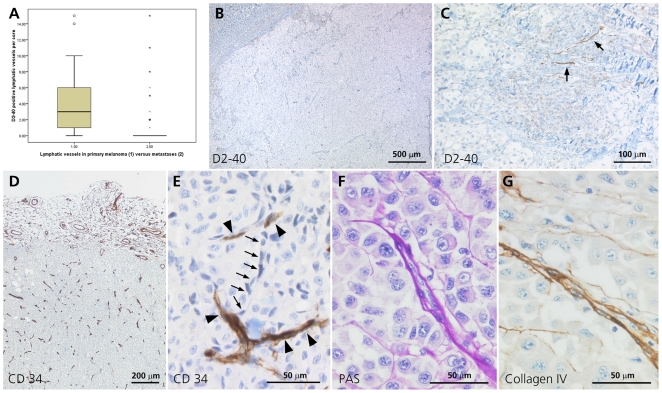
Human melanoma lymph node metastases (entirely embedded). (A) Box plot comparison of lymphatic vessels (number of D2–40 positive vessels per core) in primary versus metastatic melanoma. * One case, * two cases. (B) Absence of D2–40 staining within metastatic tissue showing no lymphatic vessels (×30). (C) D2–40 positivity shows dilated lymphatic vessels present at the tumor/lymphatic parenchyma interface (arrow, ×150). (D) CD34 staining shows important blood micro-neovascularisation within metastases. (×50). (E) CD34-positive blood microvessels (arrowhead) associated with CD34–negative cells showing vascular mimicry (arrow, ×400). (F) and (G) Cells of vascular mimicries embedded in a PAS (F) and collagen IV (G) positive laminina (arrow, ×320)).

We investigated 127 melanoma metastases (21 lymph nodes and 106 other sites) and 49 primary melanomas for vascularisation. The number of vessels was counted per core. There were significantly more D2–40 positive lymphatic vessels in primary melanoma than in melanoma metastases (p<0.0001; Figure 1A). Only 15 of 127 (12%) metastases had lymphatic vessels whereas 39 of 49 (80%) primary melanomas were positive for lymphatic vessels. Metastases had a mean of 0.6 lymphatic vessels per core (median 0; range 0–15) in contrast to primary tumors with a mean of four lymphatic vessels per core (median 3; range 0–15; Table 1). Analyzing a separate collection of 48 complete paraffin-embedded metastases (35 lymph node and 13 organ metastases) confirmed negativity for lymphatic vessels. 18 of 48 (38%) metastases were completely negative for lymphatic vessels, while 23 (48%) showed foci of dilated lymphatic vessels at the interphase to normal organ parenchyma. All but three of these were lymph node metastases (Figures 1B, C). Only seven (14%) had few lymphatic foci within the metastatic body. However, normal skin 0,5 cm adjacent to the primary melanoma showed an average of 8 vessels per mm^2^ which is congruent with the literature [31]. In contrast lymph node tissue 0,5 cm adjacent to the metastases showed no lymphatic vessels. However, in all 48 cases blood neovascularization was prominent. The lymph node metastases comprised 20 macro- and 15 micro-metastases (<2 mm). Congruent with Folkman's findings [32] we found neither lymphatic nor blood-micro-vessels in micro-metastases.

**Table 1:** Blood and lymphatic vessels per TMA core (0,28 mm^2^) in primary and metastatic melanoma.

	Primary melanomas	Metastases of melanoma	P-value
Sample number	49	127	
Lymphatic vessels (D2–40)	4±4	0.6±2	<0.0001
Blood vessels (CD34)	20±14	15±14	0.01

± Mean ± SD.

There were significantly more blood than lymphatic microvessels in primary melanomas (p<0.0001) as well as in metastases (p<0.0001). In addition primary melanomas had significantly more blood vessels than metastases (p = 0.012). Primary melanomas had an average of 20 blood vessels per core (median 19; range 1–60) and metastases had an average of 15 blood vessels (median 11; range 0–69) per core (Table 1). There was no statistically significant difference in vessel density or composition between lymph node and organ metastases. Interestingly, there was a virtual homogenous distribution of blood neovascularization over cross section of metastases. In large metastases there was no difference between central and peripheral regions. We could not find blood vessel concentrations at the interface with normal lymphatic tissue as found for lymphatic vessels (Figure 1D). Glut-1 expression in melanoma cells is an indicator of a hypoxic environment. We analyzed Glut-1 expression semi quantitatively and differentiated between Glut-1 expression in less or more than 50% of melanoma cells per core. There was a significant difference between primary melanoma and metastases (p = 0.002). 45 of 127 (35%) melanoma metastases showed Glut-1 expression in more than half of the cells whereas this was true for only 6 of 49 (12%) primary melanomas. A significant inverse correlation between blood vessel number and Glut-1 expression in melanoma (p = 0.0002) indicates the functionality of blood neovascularization.

### Melanoma cells participate in neovascularization during tumorigenesis

**Figure 2:**
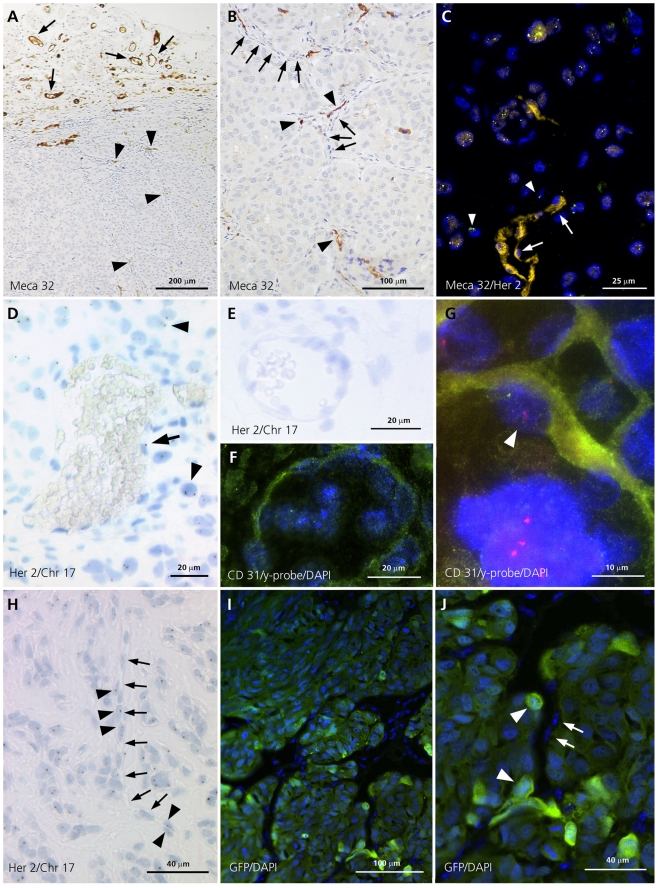
Melanoma xenograft tissues. (A) Mouse vessel-specific MECA32 staining shows micro-neovascularisation within melanoma xenografts (arrowhead, ×100) and positive vessels of the adjacent soft tissue (arrow). (B) Vascular mimicries negative for MECA32 (arrow) associated with and connecting MECA32-positive vessels (arrowhead, ×175). (C) Interphase FISH for human chromosome 17 and HER-2 combined with immunofluorescence staining for the mouse-specific vessel marker MECA32: Tumor capillaries with endothelial cells positive for MECA32 (yellow) and negative for HER-2 (arrow) as well as endothelial-like cell with negativity for MECA32 and positivity for human chromosome 17 and HER-2 (arrowhead, green; ×500). (D) and (G) Melanoma tumor cell-derived endothelial-like cells: (D) Capillary endothelial-like cell positive for human chromosome 17 and HER-2 (arrow) and positive internal control of tumor cells (arrowhead, ×500. (E) and (F) Negative internal controls of slide D and G. Melanoma xenograft adjacent soft tissue with mouse capillaries and fibroblasts negative for human chromosome 17_HER-2 probe (E, ×500) and negative for FISH y probe and CD31 (F, ×700). (G) Interphase FISH for human y-probe combined with immunofluorescence staining for the human-specific vessel marker CD31: Vessel endothelial-like cell positive for the Y chromosome (red) and CD31 (arrowhead, green; ×1000). (H) Vascular mimicries (arrow): Endothelial-like cells positive for human chromosome 17 and HER-2 (arrowhead, ×300). (K) and (L) Green fluorescent protein labelled melanoma cell lines: Melanoma cells (arrowhead, green) and endothelial cells (arrow) contributing to complex branching neo-vascularisation. (K ×200; L ×500).

**Figure 3:**
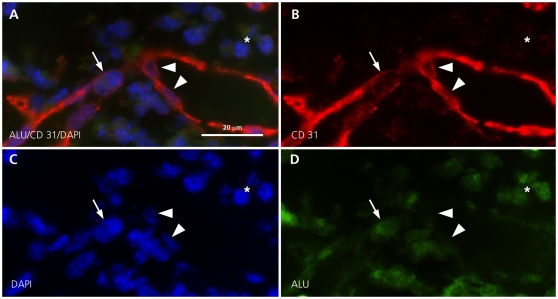
FICTION for human ALU-repeat probe and CD31 in melanoma xenograft tissue. Melanoma derived endothelial-like cells of tumor capillaries with nuclear positivity for the human-DNA specific ALU-repeat sequences (A, D arrow, green, ×700) and cytoplasmic positivity for CD31 (A, B, arrow, red, ×700). Mouse derived endothelial cells with negativity for the human-DNA specific ALU-repeat sequences (D arrow head) and cytoplasmic positivity for CD31 (B arrow head, red). Internal control with nuclear positivity for the human-DNA specific ALU-repeat sequences A, D) and negativity for CD31 in melanoma cells (A, B, asterix).

We investigated six xenografts of human melanoma cell lines labelled with p75 and sorted by FACS as described in material and methods for MECA32, a marker specific for mouse vessel endothelia. There was significant neovascularization within the tumor (Figures 2A, B). Most endothelial cells surrounding microvessels were MECA32 positive, indicating their mouse origin. However, fluorescence double-labelling showed that some were negative for MECA32 and positive for human chromosome 17 and HER-2, indicating their melanoma origin (Figure 2C). As they have the same staining properties and a similar endothelial morphology as melanoma cells of VMs we introduced for melanoma cells in VMs as well as for melanoma cells contributing to capillaries the term endothelial-like cell. For quantifying the percentage of endothelial-like tumor derived cells contributing to capillaries, chromogenic *in situ* hybridization for HER-2 gene probe and centromeric probe for chromosome 17 was used (Figure 2D). Capillaries and fibroblasts of tumor adjacent soft tissue were used as a negative internal control (Figure 2E). We identified 6% of all capillary lining cells as endothelial-like cells. Endothelial-like cells of VMs are not included in this account. In addition, tumor cell contribution to neovascularization was also confirmed by combined in situ- and immunofluorescence labelling in the mouse model displaying double-positivity of endothelial-like cells for the vessel marker CD31 and the human Y-chromosome (Figure 2G) as well as for CD31 and the human-DNA specific ALU-repeat sequences (Figure 3A–D). Mouse vessels of adjacent tissues served as internal negative controls (2F).

Table 2 overviews all experiments confirming the melanoma cells contribution to neovascularisation

**Table 2:** Overview of melanoma xenograft experiments used for illustrating melanoma cells participation in neovascularisation.

Method	Result	Shown in figure
Interphase FISH for human chromosome 17 and HER-2 combined with immunofluorescence staining for the mouse-specific vessel marker MECA32:	Capillary lining cells (endothelial-like cells) negative for MECA32 and positive for human chromosome 17 and HER-2	2C
Chromogenic *in situ* hybridization for human centromeric chromosome 17 and HER-2 gene probe	6% of all capillary lining cells (endothelial-like cells) are positive	2D
Interphase FISH for human y-probe combined with immunofluorescence staining for the human-specific vessel marker CD31	Endothelial-like cells positive for the Y chromosome and CD31	2G
Interphase FISH for the human DNA specific ALU-repeat combined with immunofluorescence staining for CD31	Endothelial-like cells positive for the human-DNA specific ALU-repeat sequences and CD31	3A–D

### Vascular mimicries in human metastases and xenograft tumors

**Figure 4:**
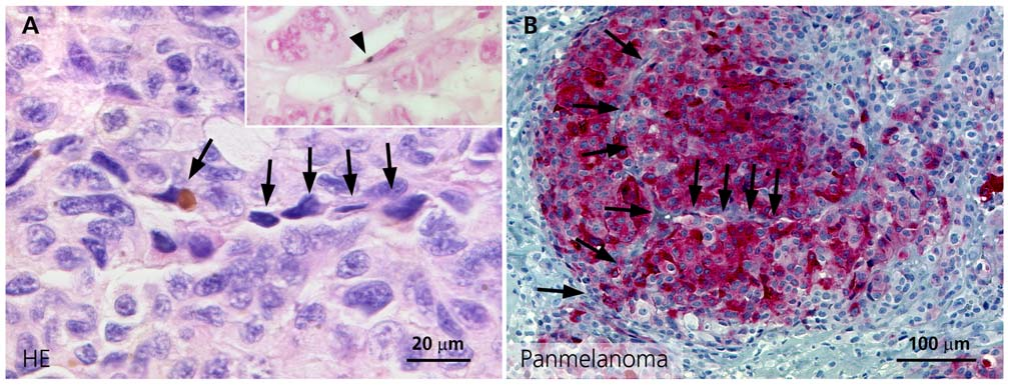
Human melanoma lymph node metastases (entirely embedded). (A) and (B) Vascular mimicries (arrow): (A) Melanoma cells with endothelial-like morphology, condensed nuclei, minimal Masson Fontana positive (inset) pigmentation (H&E, ×500), (B) mostly negative for PanMelanoma (red) (×150).

Vascular mimicries (VM) were observed in both human metastases (Figures 1E, F, G, 4A, B) and mouse xenografts (Figure 2B, H). Melanoma cells contributing to VM were embedded in a PAS (1F) and collagen IV (1G) positive extracellular matrix, were small with endothelial-like cell morphology and highly condensed nuclei (Figures 1E, F, G). Most of these cells were negative for PanMelanoma (HMB-45 and MART-1). However, some contained melanin (Masson Fontana positive) or were positive for PanMelanoma (Figures 4A, B) indicating their melanocytic origin. Most importantly, VM in xenografts were positive for the chromosome 17 and HER-2 probe (Figure 2H) and GFP fluorescence labelling, which proofed their melanoma derivation (Figure 2 I, J).

In contrast to their endothelial-like morphology, they were negative for CD31, CD34 and D2–40. In xenografts tumor cell VM were associated with MECA32-positive mouse derived microvessels (Figure 2B) whereas in human melanoma metastases they associated and connected with CD34-positive blood vessels (Figure 1E).

## Discussion

The establishment of metastases is a complex process, which includes angiogenic development. Several studies have shown that increased lymphatic density in primary melanomas is associated with a higher incidence of regional lymph node metastases [5], [33]. To date there are few studies analyzing neovascularization in human cancers and these are mostly restricted to breast cancer [6], [34], [35], [36]. In our study, we examined 175 melanoma metastases for lymphatic and blood vessel formation using immunohistochemistry. We found significantly more lymphatic vasculature in primary than in metastatic melanomas (p<0.0001).

In contrast to the lymphangiogenesis seen in primary lesions, there were few lymphatic vessels observed within lymph node and organ metastases. The few lymphatic vessels identified were mostly found in foci at the metastatic lymph node interphase, or in the lymph node parenchyma close to metastases, and these were often angiectatic (Figures 1B, C). Our histopathological observations correspond well with van den Eynden's reports on breast cancer [34]. In melanoma metastases lymphatic foci have also been reported, however no information on their *in situ* distribution was provided [6].

However, we observed a significant anatomical difference in lymphatic density with a mean of 8 lymphatic vessels in normal skin 0,5 cm adjacent to the primary melanoma and absence in lymph node tissue 0,5 cm adjacent to the metastases. However, despite those anatomical differences, there still remained a significant difference in lymphatic vascularization between primary melanoma and adjacent normal tissue indicating the lymphatic induction potential of primary melanoma, which was almost absent in metastases. Lymphatic induction in primary melanoma and its prognostic relevance is well documented. In addition lymphatic induction is also a part of regression in primary melanoma [31], [33].

Melanoma metastases presented a highly complex neovascularization with immunoreactivity for CD34 and negativity for D2–40. Furthermore, the highly significant inverse correlation between expression of Glut-1 (implying local hypoxia) and the presence of micro vessels suggests that hypoxia is a major driver (p<0.0001; Figure 1A). Interestingly there was a homogenous distribution of micro blood vessels over whole metastases. In terms of their structure, we observed that these tumor vessels are abnormal as their walls have numerous ‘openings’. Ultrastructure studies of tumor vessels have shown endothelial fenestrae, transcellular holes, widened interendothelial junctions, and discontinuous or absent basement membranes. Such defects are known to make tumor vessels leaky [37] [38]. Increasing neovascularization with leaky vessels may contribute to hematogenous spread. The vascular endothelial growth factor (VEGF), predominantly contributed by tumor cells is a major stimulator of angiogenesis [39]. Considering the tremendous neovascularisation of melanoma metastases, it is not surprising that inhibition of the VEGF signalling pathway has shown promising therapeutic benefits. Unfortunately, adaptive tumor responses are often observed, indicating the need for further understanding of VEGF regulation. Recently, a novel G protein-coupled receptor, GPR56, which inhibits VEGF production from the melanoma cell lines [40], as well as heparanase, which induces VEGF, were reported as possible tumor targets for influencing VGEF expression [41]. In contrast, one could speculate that anti-angiogenic therapies result in hypoxic condition and therefore benefit the growing of VM which represent the aggressive invasive form of melanoma.

Similar blood neovascularization was found in xenografts of human melanoma cell cultures. These vessels were mostly positive for the mouse-specific vessel marker MECA32. However, about 6% of endothelial-like cells were negative for this marker. We propose that these MECA32-negative cells are tumor-derived because they are positive for the human chromosome 17 and HER-2 (Figure 2 D), are positive for the Y-chromosome and CD31 in xenotransplantations of male melanoma patient cell lines in female mice (Figure 2 G), are positive for the ALU-repeat and CD31 (Figure 3 A–D) and they express transfected green fluorescent protein (Figure 2 I, J). Taken together, we conclude that melanoma cells acquire vascular endothelial-like morphology and are incorporated in capillaries and thus actively contribute to the establishment of neovascularization [42]. These features are in congruence with findings from the laboratory of Mary Hendrix [23]. This group injected aggressive melanoma cells containing a fluorescent label into the ischemic hind limbs of nude mice, which were re-perfused five days later. Histological cross-sections of newly formed vasculature in re-perfused limbs showed human melanoma cells adjacent to and overlapping with mouse endothelial cells in a linear arrangement.

Cells involved in VM express genes associated with embryonic stem cells including those associated with primordial vascular development [14], [19], [20], [21], [43]. In a recent study, we have provided evidence for molecular plasticity in melanoma switching between proliferative and invasive phenotypes [13]. Culturing invasive phenotype melanoma cells on matrigel causes them to adopt VM morphologies. In addition, Hess et al have shown that epithelial cell kinase is a critical molecular determinant for VM in aggressive melanoma cells. Inhibition of tyrosine phosphorylation abrogates the ability of the aggressive melanoma cells to form VMs [21]. Therefore we postulate that VM tumor cells represent the invasive phenotype *in vivo*. Very recently, two independent groups revealed that a proportion of the endothelial cells of blood vessels in glioblastoma originate from the tumour cell population as proven by tumor specific genetic alterations [25], [26].

Approximately one million tumor cells are shed per g of tumor per day [44]. Chang and Maniotis have shown in colon cancer and melanoma the functional activity of VM and their implication for tumor shedding [14], [22]. Our results indicate that the melanoma lymph node metastases with tremendous neovascularization, VM and leaky vessels are a direct contributor to hematogenous spread. The low percentage (6%) of tumor cells directly contributing to the capillary network is interesting concerning tumor cell plasticity and tumor cell niche aspects. Looking at the high number of tumor shedding, they at first seem irrelevant. However, they have a remarkable plastic capacity and by lack of melanocytic differentiation, antigens escape normal immune surveillance. Therefore, their influence in metastatic disease or tumor relapse should not be underestimated.

In summary, we found a complex neovascularization consisting of VMs and leaky blood micro vessels in human melanoma biopsies and melanoma xenografts. In addition, melanoma cells contributed to tumor capillaries.

## References

[1] Morton DL, Thompson JF, Cochran AJ, Mozzillo N, Elashoff R (2006). Sentinel-node biopsy or nodal observation in melanoma.. N Engl J Med.

[2] Balch CM, Soong SJ, Gershenwald JE, Thompson JF, Reintgen DS (2001). Prognostic factors analysis of 17,600 melanoma patients: validation of the American Joint Committee on Cancer melanoma staging system.. J Clin Oncol.

[3] Skobe M, Hawighorst T, Jackson DG, Prevo R, Janes L (2001). Induction of tumor lymphangiogenesis by VEGF-C promotes breast cancer metastasis.. Nat Med.

[4] Stacker SA, Farnsworth RH, Karnezis T, Shayan R, Smith DP (2007). Molecular pathways for lymphangiogenesis and their role in human disease.. Novartis Found Symp.

[5] Tobler NE, Detmar M (2006). Tumor and lymph node lymphangiogenesis–impact on cancer metastasis.. J Leukoc Biol.

[6] Dadras SS, Lange-Asschenfeldt B, Velasco P, Nguyen L, Vora A (2005). Tumor lymphangiogenesis predicts melanoma metastasis to sentinel lymph nodes.. Mod Pathol.

[7] Qian CN, Berghuis B, Tsarfaty G, Bruch M, Kort EJ (2006). Preparing the “soil”: the primary tumor induces vasculature reorganization in the sentinel lymph node before the arrival of metastatic cancer cells.. Cancer Res.

[8] Harrell MI, Iritani BM, Ruddell A (2007). Tumor-induced sentinel lymph node lymphangiogenesis and increased lymph flow precede melanoma metastasis.. Am J Pathol.

[9] Hirakawa S, Kodama S, Kunstfeld R, Kajiya K, Brown LF (2005). VEGF-A induces tumor and sentinel lymph node lymphangiogenesis and promotes lymphatic metastasis.. J Exp Med.

[10] Hirakawa S, Fujii S, Kajiya K, Yano K, Detmar M (2005). Vascular endothelial growth factor promotes sensitivity to ultraviolet B-induced cutaneous photodamage.. Blood.

[11] Bittner M, Meltzer P, Chen Y, Jiang Y, Seftor E (2000). Molecular classification of cutaneous malignant melanoma by gene expression profiling.. Nature.

[12] Hoek KS, Schlegel NC, Brafford P, Sucker A, Ugurel S (2006). Metastatic potential of melanomas defined by specific gene expression profiles with no BRAF signature.. Pigment Cell Res.

[13] Hoek KS, Eichhoff OM, Schlegel NC, Dobbeling U, Kobert N (2008). In vivo switching of human melanoma cells between proliferative and invasive states.. Cancer Res.

[14] Maniotis AJ, Folberg R, Hess A, Seftor EA, Gardner LM (1999). Vascular channel formation by human melanoma cells in vivo and in vitro: vasculogenic mimicry.. Am J Pathol.

[15] Shirakawa K, Kobayashi H, Heike Y, Kawamoto S, Brechbiel MW (2002). Hemodynamics in vasculogenic mimicry and angiogenesis of inflammatory breast cancer xenograft.. Cancer Res.

[16] Sun B, Zhang S, Zhao X, Zhang W, Hao X (2004). Vasculogenic mimicry is associated with poor survival in patients with mesothelial sarcomas and alveolar rhabdomyosarcomas.. Int J Oncol.

[17] Sharma N, Seftor RE, Seftor EA, Gruman LM, Heidger PM (2002). Prostatic tumor cell plasticity involves cooperative interactions of distinct phenotypic subpopulations: role in vasculogenic mimicry.. Prostate.

[18] Zhang S, Zhang D, Sun B (2007). Vasculogenic mimicry: current status and future prospects.. Cancer Lett.

[19] Seftor EA, Meltzer PS, Kirschmann DA, Pe'er J, Maniotis AJ (2002). Molecular determinants of human uveal melanoma invasion and metastasis.. Clin Exp Metastasis.

[20] Warso MA, Maniotis AJ, Chen X, Majumdar D, Patel MK (2001). Prognostic significance of periodic acid-Schiff-positive patterns in primary cutaneous melanoma.. Clin Cancer Res.

[21] Hess AR, Seftor EA, Gardner LM, Carles-Kinch K, Schneider GB (2001). Molecular regulation of tumor cell vasculogenic mimicry by tyrosine phosphorylation: role of epithelial cell kinase (Eck/EphA2).. Cancer Res.

[22] Chang YS, di Tomaso E, McDonald DM, Jones R, Jain RK (2000). Mosaic blood vessels in tumors: frequency of cancer cells in contact with flowing blood.. Proc Natl Acad Sci U S A.

[23] Hendrix MJ, Seftor RE, Seftor EA, Gruman LM, Lee LM (2002). Transendothelial function of human metastatic melanoma cells: role of the microenvironment in cell-fate determination.. Cancer Res.

[24] Butler TP, Gullino PM (1975). Quantitation of cell shedding into efferent blood of mammary adenocarcinoma.. Cancer Res.

[25] Ricci-Vitiani L, Pallini R, Biffoni M, Todaro M, Invernici G (2010). Tumour vascularization via endothelial differentiation of glioblastoma stem-like cells.. Nature.

[26] Wang R, Chadalavada K, Wilshire J, Kowalik U, Hovinga KE (2010). Glioblastoma stem-like cells give rise to tumour endothelium.. Nature.

[27] Kononen J, Bubendorf L, Kallioniemi A, Barlund M, Schraml P (1998). Tissue microarrays for high-throughput molecular profiling of tumor specimens.. Nat Med.

[28] Civenni G, Walter A, Kobert N, Mihic-Probst D, Zipser M (2011). Human CD271-Positive Melanoma Stem Cells Associated with Metastasis Establish Tumor Heterogeneity and Long-Term Growth.. Cancer Res.

[29] Graham FL, Smiley J, Russell WC, Nairn R (1977). Characteristics of a human cell line transformed by DNA from human adenovirus type 5.. J Gen Virol.

[30] Follenzi A, Naldini L (2002). Generation of HIV-1 derived lentiviral vectors.. Methods Enzymol.

[31] Yun SJ, Gimotty PA, Hwang WT, Dawson P, Van Belle P (2011). High lymphatic vessel density and lymphatic invasion underlie the adverse prognostic effect of radial growth phase regression in melanoma.. Am J Surg Pathol.

[32] Folkman J, Kalluri R (2004). Cancer without disease.. Nature.

[33] Dadras SS, Paul T, Bertoncini J, Brown LF, Muzikansky A (2003). Tumor lymphangiogenesis: a novel prognostic indicator for cutaneous melanoma metastasis and survival.. Am J Pathol.

[34] Van den Eynden GG, Van der Auwera I, Van Laere SJ, Huygelen V, Colpaert CG (2006). Induction of lymphangiogenesis in and around axillary lymph node metastases of patients with breast cancer.. Br J Cancer.

[35] Van den Eynden GG, Vandenberghe MK, van Dam PJ, Colpaert CG, van Dam P (2007). Increased sentinel lymph node lymphangiogenesis is associated with nonsentinel axillary lymph node involvement in breast cancer patients with a positive sentinel node.. Clin Cancer Res.

[36] Guidi AJ, Berry DA, Broadwater G, Perloff M, Norton L (2000). Association of angiogenesis in lymph node metastases with outcome of breast cancer.. J Natl Cancer Inst.

[37] Hobbs SK, Monsky WL, Yuan F, Roberts WG, Griffith L (1998). Regulation of transport pathways in tumor vessels: role of tumor type and microenvironment.. Proc Natl Acad Sci U S A.

[38] Hashizume H, Baluk P, Morikawa S, McLean JW, Thurston G (2000). Openings between defective endothelial cells explain tumor vessel leakiness.. Am J Pathol.

[39] Roskoski R (2007). Vascular endothelial growth factor (VEGF) signaling in tumor progression.. Crit Rev Oncol Hematol.

[40] Yang L, Chen G, Mohanty S, Scott G, Fazal F (2011). GPR56 Regulates VEGF Production and Angiogenesis during Melanoma Progression.. Cancer Res.

[41] Luan Q, Sun J, Li C, Zhang G, Lv Y (2011). Mutual enhancement between heparanase and vascular endothelial growth factor: A novel mechanism for melanoma progression.. Cancer Lett.

[42] Sommer L (2005). Checkpoints of melanocyte stem cell development.. Sci STKE.

[43] Folberg R, Rummelt V, Parys-Van Ginderdeuren R, Hwang T, Woolson RF (1993). The prognostic value of tumor blood vessel morphology in primary uveal melanoma.. Ophthalmology.

[44] Liotta LA, Kleinerman J, Saidel GM (1974). Quantitative relationships of intravascular tumor cells, tumor vessels, and pulmonary metastases following tumor implantation.. Cancer Res.

